# Why Should DNA Topoisomerase I Have a Scaffold Activity?

**DOI:** 10.3390/biology10030190

**Published:** 2021-03-03

**Authors:** Francesca Di Felice, Giorgio Camilloni

**Affiliations:** Dipartimento di Biologia e Biotecnologie, Università di Roma Sapienza, 00185 Rome, Italy; fdifel@gmail.com

**Keywords:** DNA topoisomerase 1, gene expression, *S. cerevisiae*, Sir2p, rDNA

## Abstract

**Simple Summary:**

DNA topoisomerases are enzymes responsible for controlling DNA topology. Their activity consists of relaxing the supercoiling that is derived from the basic processes that DNA undergoes, such as replication, transcription, and recombination. DNA topoisomerase actions have been observed in all organisms that have DNA as their genetic material. Although they are mainly involved in DNA relaxation, some observations show that type IB DNA topoisomerases are also involved in other processes, such as splicing, and have a role in promoting DNA transcription without using their catalytic activity. In this review, we describe the additional capacity of the DNA topoisomerase IB, beyond the main one that releases torsional stress by its catalytic activity, to act as a scaffold protein able to recruit several factors needed for transcription and regulation of gene expression.

**Abstract:**

Since the early 1990s, in vitro studies have demonstrated that DNA topoisomerase I promotes RNA polymerase II transcription, acting as a cofactor, regardless of its catalytic activity. Recent studies, carried in vivo, using yeast as a model system, also demonstrate that DNA topoisomerase I is able to recruit, without the involvement of its catalytic activity, the Sir2p deacetylase on ribosomal genes thus contributes to achieve their silencing. In this review, the DNA topoisomerase I capability, acting as a scaffold protein, as well as its involvement and role in several macromolecular complexes, will be discussed, in light of several observations reported in the literature, pointing out how its role goes far beyond its well-known ability to relax DNA.

## 1. Introduction

To date, the only function of DNA topoisomerases taken into consideration has been to solve topological problems from different DNA transactions. The acquirement of increasingly refined molecular and biochemical techniques (from the yeast two-hybrid assay up to the most recent interactomics) highlights the extreme ability of topoisomerase IB in interacting with several other protein factors, as well as its involvement in some processes that do not require its catalytic activity. In this review, we focused our attention on the DNA topoisomerase IB scaffold activity as an additional noteworthy role.

## 2. Canonical Activities of Topo I

### 2.1. DNA Structure and the Need to Evolve Enzymes for Solving Its Topological Problems

The need to evolve specific enzymes, capable of altering the topological state of DNA, is derived from the very nature of this nucleic acid. The two strands by which DNA is constituted run in pair, yielding a right-ended double helix, while being wrapped together in a plectonemic way. This implies that, in a circular covalently closed DNA molecule, as well as in a linear one with blocked ends, topological properties are well defined. Therefore, as long as the integrity of one of the two strands is maintained, any observed topological alteration can only consist of interconversions between T and W parameters, T being the twist number (which represents the winding state of the double helix), and W the writhe number (which represents the supercoiling state of the double helix) linked by the well-known relation L = T + W proposed by Fuller [1]. This equation describes how T alterations will produce compensatory W changes and vice versa, since the L factor of Fuller’s equation cannot be modified. In fact, L (linking number, number of times that one filament crosses with the other) cannot change as long as one of the two strands is not interrupted.

Since all of the DNA transactions go through local alterations of the topological state of the DNA (for example the local T, which is strongly altered when the two DNA strands are separated), with consequences on the specific process (replication or transcription progression for example), the intervention of specific enzymes that are able to solve these problems—through the relaxation of the superhelix—is of extreme importance.

### 2.2. DNA Topoisomerases

DNA topoisomerases are enzymes capable of changing the topology of DNA by breaking and rejoining one (type I DNA topoisomerases) or two (type II DNA topoisomerases) strands of the double helix. These enzymes are present in all organisms that possess DNA as genetic material and their function is mostly essential for Topo II, while in some organisms, such as *Saccharomyces cerevisiae,* the deletion of the topoisomerase I gene (*TOP1*) is tolerated [2]. However, it has been observed that, in higher eukaryotes, Topo I is an essential and necessary enzyme for the embryonic development of Drosophila [3] and mouse [4]. The distinction into different types of these enzymes mainly depends on the differences in their mechanisms of action, referred to their interventions on one or both DNA strands. The covalent intermediate obtained during the reaction with the formation of a phosphotyrosine bond and the generation of a free 5’- or 3’-OH end on the DNA is another element by which different topoisomerases can be distinguished, as well as the type of supercoiling they are able to resolve (whether positive or negative) and the cofactor requirements. Table 1 reports the general subdivision of the DNA topoisomerases depending on their main characteristics.

Excellent reviews describing the structure, reaction, and function of topoisomerases have been published over the years [5,6,7,8]. Here, we will focus on an emerging new role for the eukaryotic DNA topoisomerase IB (hereafter, called Topo I) as a recruiting factor that can, in turn, be recruited within functional complexes, regardless of its catalytic activity by which it can modify DNA topology.

### 2.3. DNA Topoisomerase IB

Like all DNA topoisomerases, Topo IB is capable of altering the linking number of a DNA molecule. This activity is made possible by a nucleophilic attack that the tyrosine residue of the catalytic site of the enzyme produces on the phosphodiester bond of one of the two DNA strands. The transesterification reaction that follows leads to the formation of a phosphotyrosine bond and the generation of a 5’-OH end on the interrupted strand. Starting from this covalent intermediate a controlled rotation of the broken DNA strands occurs. Now, the 5’-OH end in turn carries a nucleophilic attack to the existing phosphor-tyrosine bond, giving a new transesterification reaction leading to the reconstitution of the phosphodiester bond, accompanied by the release of the enzyme and the reconstitution of an intact DNA molecule. The reaction does not involve any energy consumption, as only the transfer reactions of bonds between DNA and enzyme take place. The net result is relaxation of both negatively and positively supercoiled DNA molecules. 

This intervention obtained through the changing of a DNA linking number by interrupting, relaxing, and finally rejoining DNA strands, is required, as DNA continuously undergoes processes, such as replication, transcription, and recombination, and all of these processes determine topological alterations. 

Champoux and Dulbecco [9] hypothesized DNA topoisomerase as a swivel element during the replication, due to the DNA polymerases molecules progression that keep strands separated during DNA synthesis, with increasing of torsional stress both upstream and downstream the replication fork, as a consequence. Similarly, Liu and Wang proposed the model of the “twin domains” formed during transcription [10]. Implications of these observation are: (i) RNA polymerase does not proceed around DNA, but it is this latter that flows on it, and (ii) the tracking action of the separate helices generates the two twin domains, which determine positive supercoiling along transcription progression (RNA synthesis) and negative in the opposite direction (behind the RNA polymerase machinery). This model has been demonstrated both in vitro and in vivo [11,12]. Even during recombination ad repair processes, the involved helicase and DNA polymerase activities further require DNA topoisomerase to overcome the topological processes [13]. 

A series of conceptual deductions on Topo I activity has been achieved by using the highly specific drug camptothecin, which traps the enzyme once it has cleaved DNA [14], allowing the formation of a ternary complex formed by the topoisomerase I, the DNA, and the camptothecin itself, thus preventing DNA re-ligation. This property allowed the mapping of Topo I cleavage sites that are scattered along actively transcribed DNA regions, but are absent when the same regions are silent [14].

### 2.4. How Is a Region Recognized Where Topological Problems Have to Be Solved?

Two-way exists by which Topo I reaches the sites where its action is required: (i) Topo I is constantly active on DNA molecules, carrying out futile relaxation cycles, both in the presence and absence of torsional stress; (ii) Topo I itself is able to recognize and resolve the torsional stress of DNA. In support of this latter hypothesis, a series of experimental data obtained in the 1990s demonstrated how Topo I prefers to bind and react with supercoiled DNA over relaxed, both in terms of reaction and binding efficiency [14,15]. Furthermore, it has been observed in vivo that DNA damage, due to the employment of camptothecin, is more relevant in actively proliferating cells and in highly transcribed regions, suggesting that the more DNA supercoils, the more the enzyme is active [16].

## 3. Catalysis Independent Functions of Topo I

It has been proposed that Topo I also has other functions, in addition to the “topoisomerase” activity that relaxes the DNA supercoiling raised by transcription, replication, or chromatin dynamics. It has been reported that Topo I possesses a “kinase” activity that does not require the tyrosine of its active site [17]. This activity is responsible for the phosphorylation of some RNA splicing factors [18] known as Serine-Arginine (SR) proteins. These proteins constitute a conserved family of splicing factors required for both constitutive and alternative splicing, also involved in mRNA transport, translation, and decay [19]. Moreover, SR proteins have been implicated in transcription elongation and genomic stability [20]. Through its ability to phosphorylate SR proteins, Topo I modulates their activity during splicing [21] and prevents replication fork collapse by suppressing R-loop formation in an SR protein-dependent manner [22]. Therefore, the presence of both torsional stress relaxation and kinase activities in the same protein can fulfill the functions required to coordinate transcription with RNA maturation events [23]. Other observations report a non-catalytic activity of Topo I, demonstrating that Topo I is recruited on *S. cerevisiae* ribosomal genes by Fob1p; thus, contributing to the transcriptional silencing of this region, all without the support of the catalytic activity [24]. Therefore, Topo I can be recruited in certain chromatin regions through protein–protein interactions. Its ability to interact with several and various protein factors had already been observed in the 1990s by R. Sternglanz and co-workers, who demonstrated the specific interaction of Topo I with Tof1p and Tof2p factors, identified through the two-hybrid assay [25]. Yeast Tof1p belongs to the replication-pausing checkpoint complex [26]. In cooperation with Mrc1p and Csm3p proteins, Tof1p interacts with stalled replication fork due to damaged DNA and promotes DNA repair. In this context, an interaction with MCM helicase has also been reported [27]. Yeast Tof2p is involved in chromatin condensation and rDNA silencing [28]. At ribosomal DNA, Tof2p recruits cohesin ring at the replication fork barrier; thus, inhibiting unequal exchange of sister chromatids. This activity is carried out due to the interaction with the Lrs4p/Cms1p component of the Regulator of nucleolar silencing and telophase exit (RENT) complex [28]. Further interactomic studies, including global ones, have highlighted that Topo I has a very large ability to bind other protein factors and interact with different proteins. According to the updated version of BioGRID [29], human Topo I has about 1000 different partners, as well as yeast Topo I. Compared with other topoisomerases, such as Topo II or Topo III, Topo I shows a larger number of interactors. These are very high numbers when compared with those referring to proteins that act as interactors par excellence, such as histones or Tata Binding Protein (TBP) (see interactome maps on BioGRID). Even if the rate of false interactions and incorrect peptide assignments, due to protein sequence homology, could affect the quantitative estimation of interactions, the fact remains that Topo I is a highly interacting protein compared to other chromatin interacting proteins. This suggests that not only Topo I is recruited into different complexes, but it can, in turn, act as a recruiter of other elements, thus acting as a scaffold protein.

Considering this huge binding capacity of Topo IB and evaluating other literature observations, it is clear that Topo I itself can function as a scaffold. In vitro experiments [29,30,31] carried in the1990s showed how, regardless of its enzymatic activity, Topo I is able to stimulate transcription in mammalian promoters, probably through the recruitment of TBP. More recently, studies on transcriptional silencing of the ribosomal genes of *S. cerevisiae* have shown how important Topo I is for the maintenance of the silenced state of this locus, and how this function has to be addressed to its role as scaffold protein [24]. In fact, it has been shown that Topo I recruits Sir2p on the Intergenic spacer (IGS) regions of ribosomal genes to achieve their transcriptional silencing. The ability of Topo I in recruiting Sir2 lies in its N-terminal region and it is independent on its catalytic activity. Therefore, in vivo and in vitro observations suggest that Topo I may also play the role of scaffold protein, capable of both recruiting fundamental factors for the control of gene expression and being recruited. Its ability to preferentially react with supercoiled DNA represents an extremely refined way to measure transcriptional activation or trigger DNA replication and recombination. 

Given the different abilities of Topo I to participate in equally different processes, it could be considered a moonlighting protein whose multiple functions are not linked to gene fusions, RNA splicing variants, or different proteolytic fragmentations. To date, hundreds of examples of moonlighting proteins have been reported [32]. 

**Figure 1:**
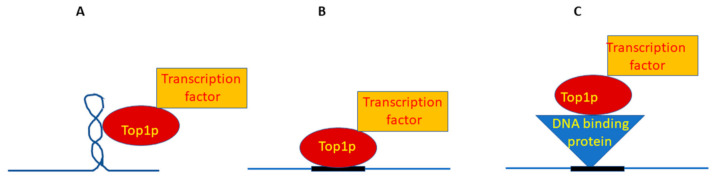
Three possible scenarios of where Topo I protein (Top1p) recruits a transcription factor. (**A**): Top1p preferentially reacts with supercoiled DNA, and in those regions, it recruits protein factors. (**B**): Top1p reacts with specific DNA sequences and recruits protein factors in these regions. (**C**): A DNA binding protein recognizes its cognate sequence, recruits Topo I, which in turn recruits a transcription factor.

We here propose three different scenarios for the Topo I intervention as a scaffold protein, depending on its recruitment on DNA. Scenario 1 (Figure 1A): DNA supercoiling attracts Topo I, known to preferentially relax supercoiled DNA [14,15], and the enzyme acts through its catalytic activity and releases torsional stress. Moreover, through its scaffolding activity, a pioneering factor promoting gene expression is recruited. Scenario 2 (Figure 1B): specific DNA sequences, such as those found in ribosomal genes [33,34] recruit Topo I, which, in turn, through its scaffolding activity, recruits the pioneering factor favoring gene expression. Scenario 3 (Figure 1C): a DNA-binding protein (such as Fob1p [35]) recruits Topo I in a given region. Through Topo I scaffolding activity, a transcriptional pioneering factor is recruited.

The hypotheses reported in Figure 1 are all based on experimental data, thus justifying how Topo I can “serve” the molecular machinery to recruit important factors to promote gene expression. The reported examples involve basic proteins, such as TBP [29,30,31], histone deacetylase (such as Sir2p) [24], or structural factors [35]; moreover, remodeling machineries or chromatin components may also be recruited according to interactome data [27]. 

How widespread are these phenomena? Are the given examples specific and particular or do they belong to a more general way of recruiting elements to the transcriptional apparatus? Further experimentation is needed to shed light on this issue in order to answer to these potential new and important questions.

## 4. Concluding Remarks

Previous in vitro and in vivo experiments have shown activities of DNA topoisomerase IB due to properties that go beyond its catalytic activity, thus suggesting that this enzyme may have additional roles, besides that of relaxing DNA. Recent in vivo studies have shown Topo I interactions with chromatin regulators, while interactomic data strongly indicate how the enzyme is able to interact with a huge number of protein partners. Therefore, the ability of Topo IB to recruit other protein factors, together with its requirement in several processes beyond its catalytic activity, makes its role as scaffold protein as important as that in DNA topology control. Future research is desirable to unveil the unknown side of this fascinating enzyme.

## Figures and Tables

**Table 1:** Eukaryotic DNA topoisomerases, types, and basic features.

Topoisomerase	Cut/s on DNA Strands	Cofactor(s) Requirement	Covalent Intermediate	Relaxation	Examples
IA	Single	Mg^++^	5′-OH	Negative supercoil	Top3α human Top3βhuman Top3 yeast
IB	Single	No	3′-OH	Negative and positive supercoil	Top1 human Top1 yeast Top1 Mitochondrial
IIA	Double	ATP, Mg^++^	5′-OH	Negative and positive supercoil	Top2 yeast Top2α human Top2β human
IIB	Double	ATP, Mg^++^	5′-OH	Negative and positive supercoil	Top VI *Arabidopsis*

## Data Availability

Data sharing not applicable.
